# Conformational stability of peroxidase from the latex of *Artocarpus lakoocha*: influence of pH, chaotropes, and temperature

**DOI:** 10.3389/fpls.2024.1341454

**Published:** 2024-02-27

**Authors:** Kirti Shila Sonkar, Manendra Pachauri, Amit Kumar, Himanshi Choudhary, Medicherla V. Jagannadham

**Affiliations:** ^1^Molecular Biology Unit, Institute of Medical Sciences, Banaras Hindu University, Varanasi, India; ^2^Department of Biochemistry, University of Delhi, New Delhi, India; ^3^Molecular Pathology Group, International Centre for Genetic Engineering and Biotechnology (ICGEB), Trieste, Italy

**Keywords:** *Artocarpus lakoocha*, circular dichroism, fluorescence spectroscopy, molten globule, peroxidase

## Abstract

The latex of the medicinal plant *Artocarpus lakoocha (A. lakoocha)*, which has been shown to have potential anti-inflammatory and wound-healing capabilities, contains a novel heme-peroxidase. This protein was subjected to activity assays, fluorescence spectroscopy, and far-UV circular dichroism to investigate its structure, dynamics, and stability. The results demonstrated the presence of three folding states: the native state (N) at neutral pH, intermediate states including molten globule (MG) at pH 2 and acid-unfolded (UA) at pH 1.5 or lower, and acid-refolded (A) at pH 0.5, along with alkaline denatured (UB) at pH 8-12 and the third denatured state (D) at GuHCl concentrations exceeding 5 M. Absorbance studies indicated the presence of loosely associated form of heme in the pH range of 1-2. The protein showed stability and structural integrity across a wide pH range (3-10), temperature (70°C), and high concentrations of GuHCl (5 M) and urea (8 M). This study is the first to report multiple ‘partially folded intermediate states’ of *A. lakoocha* peroxidase, with varying amounts of secondary structure, stability, and compactness. These results demonstrate the high stability of *A. lakoocha* peroxidase and its potential for biotechnological and industrial applications, making it a valuable model system for further studies on its structure-function relationship.

## Graphical Abstract

A peroxidase from *Artocarpus lakoocha* in its different folding states, observed under various denaturing conditions.

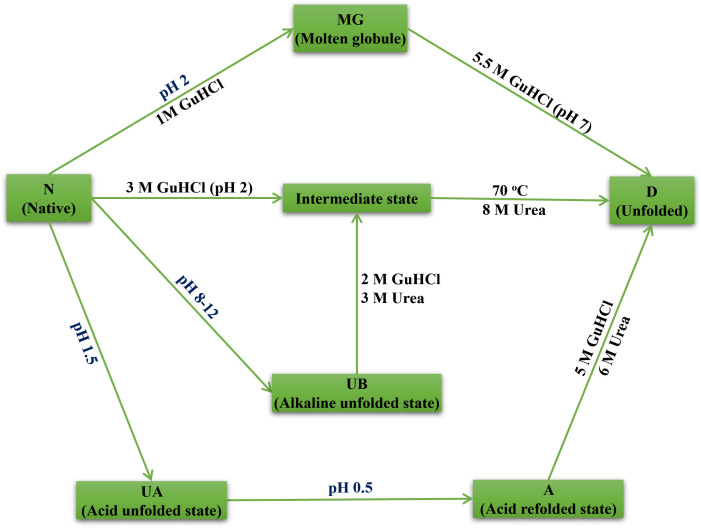

## 1 Introduction

A member of the Moraceae family, *Artocarpus lakoocha* (*A. lakoocha*) is a medicinal plant. This popular Indian medicinal plant is used to treat a wide range of illnesses, including taeniasis (Charoenlarp et al., 1989), intestinal fluke Haplorchis taichui (Wongsawad et al., 2005), and cosmetics (Theorell, 1941). For proteins to function, they must undergo significant conformational changes, which must be tracked to comprehend how proteins work. The amino acid sequence of proteins determines their folding and conformational stability, together with extrinsic biological factors including pH, temperature, and chemical denaturants (Anfinsen, 1973; Masson and Lushchekina, 2022; Sonkar et al., 2023). To predict how proteins will behave under different conditions, it is important to measure and model the thermodynamic parameters of protein folding. The protein’s folded state is stabilized by several forces, including hydrophobic, van der Waals, entropic effects, hydrogen bonding, and disulfide bonding (Eslami-Farsani et al., 2021). From unfolded chain to a functional three-dimensional state, the protein undergoes various non-native intermediate forms that are involved in various cell phenomena such as chaperone action (Uversky, 2010; Díaz-Villanueva et al., 2015), amyloid fibril formation in pathological cells (Almeida and Brito, 2020). A better understanding of protein folding pathways can explain the mechanisms of protein misfolding disorders, such as prion diseases, Parkinson’s disease, and Alzheimer’s disease Protein folding involves many intermediates between the native (N) and unfolded (U) states. Knowing these transition stages is important to understand how and when different factors act to guide the protein toward achieving three-dimensional functional structures. Molten globules (MG) are stable, partially folded states between N and U. They display initial folding events under mild stress before specific side-chain interactions form. Protein folding involves many intermediates between the native (N) and unfolded (U) states. Knowing these transition stages is important to understand how and when different factors act to guide the protein toward achieving three-dimensional functional structures. Molten globules (MG) are stable, partially folded states between N and U. They display initial folding events under mild stress before specific side-chain interactions form. They have a secondary but not tertiary structure, resulting in lower compactness than N and higher than U MGs have exposed hydrophobic surfaces that bind hydrophobic dyes, providing experimental evidence of intermediate structures (Judy and Kishore, 2019). The exact role of MGs in protein folding is unclear, and different MGs can occur in the same protein (Bychkova et al., 2022). Far-UV and near-UV CD spectroscopy are common methods to detect secondary and tertiary protein structures and are the experimental methods commonly employed to find intermediate states. Hydrophobic dye-binding assays, such as ANS (8-anilino-1-naphtalene-1-sulfonate), can measure the hydrophobic core and the solvent-exposed region of Intermediates (Dehkordi et al., 2021). This context led to the studies of the conformational stability of novel peroxidase from the latex of *A. lakoocha* plant, extending our previous work on its folding characteristics (Sonkar et al., 2015). In our previous study, we isolated a novel peroxidase from *A. lakoocha plant*, a stable heme-peroxidase with anti-inflammatory and wound-healing properties, attracting further investigation into its folding behavior. This study investigates the conformational features of *A. lakoocha* peroxidase, a 53 kDa plant enzyme with α and β structures, and its biophysical attributes, conformational stabilities, and unfolding patterns to understand its function and origins. The study investigates the conformational stability of a peroxidase under different denaturing conditions and identifies various equilibrium intermediate states with distinct stabilities, which improves our knowledge of peroxidase folding mechanisms. These studies contribute to the understanding of protein folding, which has implications for biotechnology and other scientific disciplines, by determining the stable folded state of proteins.

## 2 Materials and methods

### 2.1 Materials

According to a previously published approach, peroxidase was extracted from fresh latex of *Artocarpus lakoocha* plants (Sonkar et al., 2015). ANS (8-anilino-1-naphthalene sulphonate, CAS 82-76-8) and GuHCl (Guanidine hydrochloride, CAS 50-01-1) HCl (Hydrochloric acid, CAS 7647-01-0) and KCl (Potassium chloride, CAS 7447-40-7) were purchased from Sigma-Alderich, USA. All other used reagents were of analytical grade. Before performing spectroscopic measurements, the protein samples were centrifuged, and the protein concentration and buffer pH were measured.

### 2.2 Methods

#### 2.2.1 Absorbance spectroscopy

A Beckman DU-640B spectrophotometer with a constant-temperature cell holder was used for the spectroscopic analysis. For all absorbance measurements, a protein concentration of 1 mM was utilized. An extinction coefficient (Ɛ^1%^_280nm_) of 16.3 M^-1^ cm^-1^ was used in spectrophotometric analysis to measure the enzyme’s concentration. A JASCO J-815 spectropolarimeter (JASCO Corporation, Tokyo, Japan) fitted with a constant temperature cell holder was used to measure far-UV circular dichroism (CD). Ammonium (+)-10-camphor sulfonate was used to calibrate the device, and conformational changes in the peroxidase’s secondary structure were seen in the 200-260 nm range. Mean residue ellipticity was determined by subtracting the relevant blanks and applying the equation given by Balasubramanian et al (Balasubramanian and Kumar, 1976) as


[θ] MRW=θobs × MRW/10cl

Where l is the path length in centimeters, c is the protein concentration (mg/ml), MRW stands for mean residue weight, and *θ*obs is the observed ellipticity in degrees. 112 was considered the mean weight of amino acid residues (MRW). The far-UV CD measurements were performed with sensitivity values of 1 and 2 millidegrees/cm.

#### 2.2.2 Fluorescence spectroscopy

The Eclipse Cary Varian UV-Vis spectrofluorometer (Varian Inc., Palo Alto, California, USA) was used to measure fluorescence and was connected to a Peltier temperature controller. To observe intrinsic tryptophan fluorescence, the protein was excited at 292 nm, and the emission of fluorescence was tracked between 300 and 400 nm (Adman and Jensen, 1981). *A. lakoocha* peroxidase activity was observed under different pH and chemical denaturant conditions utilizing hydrogen peroxide as a hydrogen acceptor and other substances like guaiacol as a hydrogen donor. 1 mM of peroxidase was introduced to a 50 mM acetate buffer (substrate II) containing guaiacol and 0.2 mM hydrogen peroxide (substrate I). At 470 nm, the guaiacol absorbance change rate was calculated.

#### 2.2.3 pH denaturation of A. lakoocha peroxidase

Using a variety of buffers, including KCl-HCl buffer (pH 0.5-1.5), Gly-HCl buffer (pH 2-3.5), sodium acetate buffer (pH 4.0-5.5), sodium phosphate buffer (pH 6.0-8.0), Tris-HCl buffer (pH 8.5-10.5), and Gly-NaOH buffer (pH 11-12.5), acid denaturation of peroxidase was found at a broad pH range. The buffers utilized in this investigation have a combined concentration of 50 mM. The proper buffer was mixed with a stock solution of the protein, and the mixture was incubated for 24 hr at 25°C.

#### 2.2.4 8-aniline-1-naphthalene sulfonate binding assay

A small amount of methanol does not affect the protein’s structure because *A. lakoocha* peroxidase exhibits activity and stability up to 50% methanol (Sonkar et al., 2015). ANS is extremely soluble in methanol (Hawe et al., 2008). ANS solution in methanol was made by this. For the measurement of ANS concentration, an extinction coefficient value of 5000M^−1^ cm^−1^ at 350 nm was employed. The suspension was incubated for 30 minutes in the dark at a molar ratio of 1:100 for protein to ANS. After that, the protein-ANS complex’s extrinsic fluorescence was seen by taking a 400-600 nm emission spectrum at an excitation wavelength of 380 nm. Slit widths of 10 and 5 nm were utilized for emission and excitation, respectively, with a protein concentration of 10 µM.

#### 2.2.5 Guanidine hydrochloride and urea-induced unfolding

From the solution’s refractive index, concentrations of GuHCl solutions were ascertained. Enzyme denaturation was seen at various pH levels as the chemical denaturant concentration rose. GnHCl is prepared in relevant pH buffers, pH of the solution with 6 M GnHCl was measured using a pH meter. Further, the concentration of GnHCl was determined using refractometry. After incubating for 24 hours at 25°C with the appropriate denaturant concentration, the peroxidase sample was allowed to come to equilibrium.

#### 2.2.6 Thermal unfolding of peroxidase

Specific procedures were followed to perform temperature-induced denaturation of the enzyme as the temperature increased. Before measurements are made, the enzyme samples are heated to progressively higher temperatures and maintained there for fifteen minutes. To verify that the target temperature is attained and maintained, the samples’ temperature is tracked using a thermocouple and a digital multimeter. Additionally, the samples are checked for protein aggregation using light scattering measurements, which can indicate if the proteins have undergone irreversible denaturation due to heat (Naeem et al., 2016). This method allows for the study of how temperature affects the stability and folding of the enzyme, which can provide insight into its function and potential for thermal inactivation.

#### 2.2.7 Data analysis

Plotting the denaturation curves involved determining the temperature or denaturant molarity with the fluorescence intensity ratio at the emission wavelength maxima of both the native and denatured proteins. CD spectroscopy was used to observe the denaturation curves of urea and guanidine hydrochloride (GuHCl). Using Far-UV CD spectroscopy with an acquisition range of 215-260 nm, denaturation studies were carried out. For fitting purposes, data at 222 nm was used, presuming a three-state model. This enabled it feasible to use the following Equation to determine the experimental equilibrium values of m (m_urea_ and m_GuHCl_) and Cm (concentration at which half-denaturation occurs):

This model assumes that the protein exists in three states: native monomeric state (N) or unfolded monomeric state (U), with I_1_ representing the unfolding intermediates. This is how the three-state equilibrium model is represented: $\;N\;\overset{{\text{K}}_{1}}{↔}I\;\overset{{\text{K}}_{2}}{↔}U$ The equilibrium constants for the first and second stages are, respectively, K_1_ and K_2_. The following equations describe this model’s thermodynamics:


$$Q=\;1\;+\;K_{1}\;+\;K_{1}K_{2}$$

XN= 1Q ; XI= K1Q; XU=K1 K2Q

$$\;\Delta {\text{G}}_{1}=\;\Delta {\text{G}}_{1}^{0}-m_{1\;}\text{D}=-RTlnK_{1};\;\Delta {\text{G}}_{2}=\;\Delta {\text{G}}_{2}^{0}-m_{2\;}\text{D}=-RTlnK_{2};\;\Delta {\text{G}}_{3}=\;\Delta {\text{G}}_{3}^{0}-m_{3\;}\text{D}$$

Therefore, the Equation in this instance can be expressed as


$$F\left(D\right)=\;X_{N}F_{N}\;+\;X_{1}F_{1}\;+\;X_{U}F_{U}$$

Consequently, the complete denaturation curve may be expressed using the two variables *S(D)* and *[D]* in the following way.


$$S\left(D\right)=\;\frac{S_{N}+S_{1}\;exp-\left(\Delta G_{1}^{0}-m_{1\;}D\right)+F_{U}\;exp-\left[\Delta G_{1\;}^{0}+\Delta G_{2}^{0}-(m_{1\;}+m_{2}\right)D]\;}{1+exp-\left(\Delta G_{1}^{0}-m_{1\;}D\right)+exp-\left[\Delta G_{1\;}^{0}+\Delta G_{2}^{0}-(m_{1\;}+m_{2}\right)D]}$$

experimental CD signal as a function of denaturant concentration is represented by the model’s variable S. Where the parameters for the N and U states are denoted by S_N_ and S_U_, respectively, and the parameters for the intermediate stages by S_1_. Applying this formula will provide m_1_, m_2_, and m_3_ in addition to the free energy differences between the native form and the intermediate, and unfolded states. T is the experimental temperature (298.15 K), and R is the ideal gas constant (Pacheco-García et al., 2022). X_N_, X_1_, and X_U_ are the mole fractions of the proteins in their native, intermediate, and unfolded states, respectively, and Q is the partition function. In the presence of denaturant (D), the standard free energy changes for the unfolding transition are ΔG_1_, and ΔG_2_, whereas in the absence of denaturant (D), the standard free energy changes are $\Delta {\text{G}}_{1}^{0},\text{and }\Delta {\text{G}}_{2}^{0}\;$ . The denaturant susceptibility parameters, m_1_, and m_2_ define how standard energy depends on denaturant concentration. This is the linear extrapolation method being used to analyze chemical denaturation data. This model offers a streamlined method for evaluating the cooperativity of *A. lakoocha* peroxidase’s reversible chemical unfolding.

While the two-state model assumes a two-state $N\;\overset{{\text{K}}_{\text{eq}}}{↔}U$ unfolding mechanism, where only folded and unfolded conformations are present at any point on the curve. The way Shirley (1995) analyzed unfolding transitions was applied to determine transition midpoints and assess the cooperativity or non-cooperativity of the transitions. Molar ellipticity, fluorescence emission maxima, and relative activity findings were all normalized and reported as a fraction unfolded to facilitate comparisons between the various measures. The following formula is applied for calculating the fraction unfolded (Su):


$$S_{u}=\left(S_{obs}-S_{n}\right)/\left(S_{u}-S_{n}\right)$$

Where S_n_ stands for the native protein signal, S_u_ for the corresponding signal of the denatured protein, and S_obs_ for the observed signal at a specific denaturant concentration. The linear dependency of the signal on the denaturant concentration before and after the transition is extrapolated to yield the values for S_n_ and S_u_, respectively. Denaturant blanks are used to ensure that the observed signal is specific to the peroxidase and not due to the presence of the denaturant.

## 3 Results

### 3.1 Absorbance spectroscopy

**Figure 1:**
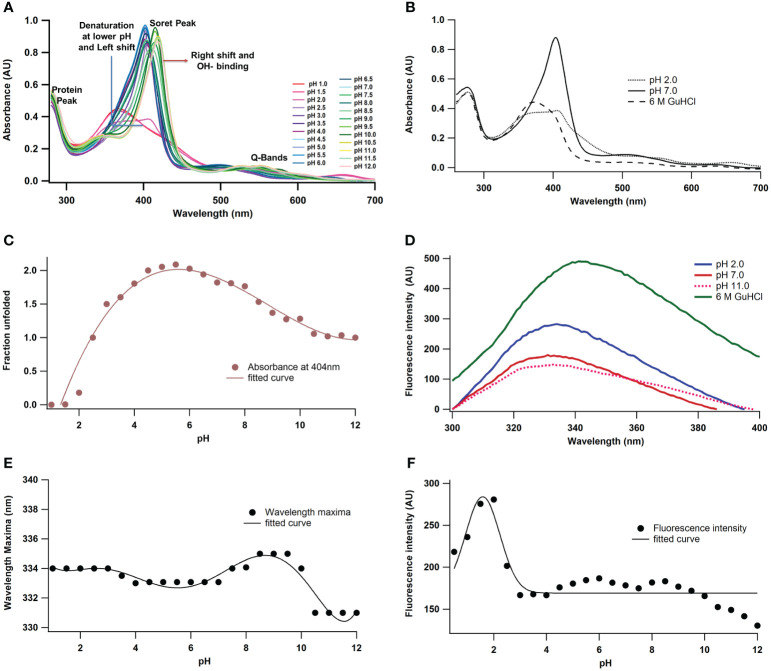
pH-induced conformational changes of *A. lakoocha* peroxidase: **(A)** UV absorbance spectra at different pH, **(B)** Spectra at pH 7.0 and 6 M GuHCl have been compared with spectra at pH 2.0 (loosely associated form of heme), **(C)** The absorbance values at 404 nm under different pH conditions., **(D)** fluorescence spectra at different pH and in 6.0M GuHCl, **(E)** fluorescence wavelength maxima, and **(F)** fluorescence intensity at different pH.

To elucidate the structure-function relationship of *A. lakoocha* peroxidase and its impact on enzymatic activity, various biophysical techniques were used. These approaches facilitate the observation of enzyme intermediates under varying pH conditions and during the denaturation process. Peroxidase’s absorbance spectra in the visible and ultraviolet (UV) areas showed four bands: the α-band at ~630 nm, the β-band at ~502 nm, the γ-band or Soret band at 404 nm, and the peroxidase absorbance spectra at 280 nm (**Figure 1A**). These bands were used as tools to investigate the functional and structural characteristics of *A. lakoocha* peroxidase, resulting in changes in their wavelength or intensity upon ligand binding or unfolding which can shed light on the alterations in the enzyme’s conformation. Substantial changes at 404 nm have been observed upon ligand binding or unfolding, indicating alterations to the enzyme’s tertiary, quaternary, or both structures as mentioned earlier (Sonkar et al., 2015). However, changes at 280 nm were not observed under native or unfolded conditions, indicating that the secondary structure of the enzyme remains unchanged. The shifts at the 404 nm wavelength are evaluated to study the conformational change of the peroxidase. To see the variations in the spectra, the absorbance spectra of *A. lakoocha* peroxidase in its native form, at pH 2, and in its unfolded condition (6 M GuHCl) (**Figure 1B**) are compared. The absorbance spectra display a soret peak at 404 nm under native conditions; this peak is absent in the unfolded state. Furthermore, **Figure 1C** displays the peroxidase absorbance maxima obtained at 404 nm under varied pH conditions, suggesting that the unfolding process causes the enzyme to undergo a conformational shift. The bell-shaped pattern indicates that the physiological pH range of the enzyme, which is 3.5 to 8.5, is where peroxidase is stable. This information is useful for understanding the stability and activity of the enzyme under different conditions.

### 3.2 Fluorescence spectroscopy

Protein fluorescence originates from tryptophan, tyrosine, and phenylalanine residues, with phenylalanine having a low quantum yield and tyrosine yield inhibited by tryptophan. Tryptophan emission spectra depend on the amino acid environment, showing red fluorescence in solvent and decreased quantum yield in quenching agents (Dehdasht-Heidari et al., 2021; Raeessi-Babaheydari et al., 2021). *A. lakoocha* peroxidase has seventeen tryptophan residues, making it a suitable protein for fluorescence quenching studies, as previously explained for native carboxypeptidase A, which has seven tryptophan residues (Mohammadi et al., 2021). **Figure 1D** shows the fluorescence spectra of peroxidase at pH 2, 11.0, and with 6 M GuHCl under native conditions. The emission maximum of peroxidase’s fluorescence spectrum in its native state occurs at 333 nm, indicating that the tryptophan residues in the enzyme are in a hydrophilic environment. The fluorescence emission maximum does not vary by acidity or alkalinity; however, in contrast to the native condition, the fluorescence intensity increases by 58% at pH 2 (178 to 280) and decreases by 17% at pH 11 (178 to 149). This suggests that variations in pH may have an impact on the tryptophan residues’ microenvironment and fluorescence properties. Complete unfolding of the protein in the presence of 6 M GuHCl results in an approximately 2-fold increase in fluorescence intensity (from 178 to 491) and a red shift of 8 nm in the emission maxima of peroxidase (from 333 nm to 341 nm), implying that the tryptophan residues unfold and meet a more hydrophobic environment, which causes the fluorescence intensity to increase and the emission maximum to shift red (Dehkordi et al., 2021). The intrinsic emission of the fluorescence scale also showed how varying pH values affected *A. lakoocha* peroxidase. **Figure 1E** displays the maximum wavelength of peroxidase with pH. The emission maximum at different pH values was not significantly altered by the transition curve, which suggested that three state transitions were involved. In the first transition, the unfolded state undergoes a redshift, with its pH decreasing from 4.0 to 2.0, with an additional red shift observed between pH 6.0 and 8.5. As the pH rises to the basic area, a third transition results in the alkaline denatured state of peroxidase occurring in the range of 9.0 to 10.5. This transition is accompanied by a blue shift of 4 nm in the fluorescence emission maximum. The non-cooperative pH-induced transition results in a little alteration in the transition maxima, as tryptophan residues appear to be exposed even in their native condition. **Figure 1F** displays variations in fluorescence intensity with pH. Two transition phases were visible in the fluorescence intensity spectra. The initial transition takes place between pH 4.0 and pH 2, transforming the native state into an acid-unfolded state. The midpoint of this transition is at pH 3.0 when there is a little increase in fluorescence intensity. Between pH 8.0 and pH 12.0, there is an incomplete second transition towards basic pH. This analysis of peroxidase’s intrinsic fluorescence spectra at various pH values sheds light on the conformational changes that the enzyme goes through and its stability at various pH levels.

### 3.3 ANS binding assay

**Figure 2:**
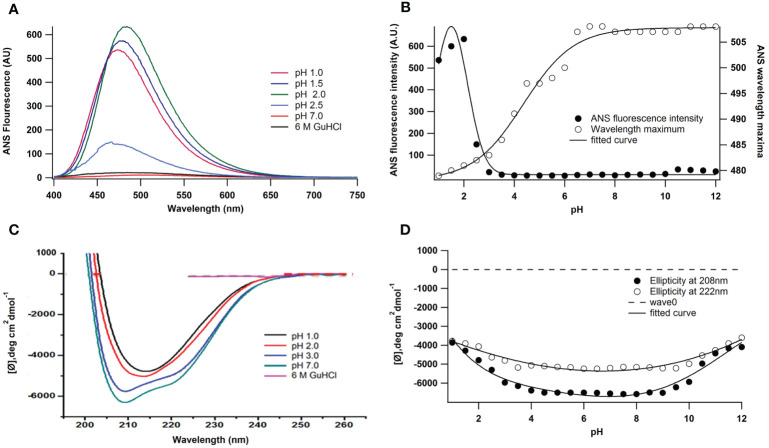
Effect of pH on the ANS fluorescence of *A. lakoocha* peroxidase: **(A)** ANS binding to peroxidase was studied at pH 1.0, pH 1.5, pH 2.0, pH 2.5, pH 7.0, and in the presence of 6M GuHCl, and **(B)** For intrinsic fluorescence measurement, samples were incubated for 24 h at 25oC. ANS emission spectra were recorded with excitation at 380nm., pH-induced conformational changes of *A lakoocha* peroxidase: **(C)** far-UV CD spectra at different pH and in 6.0 M GuHCl, and **(D)** Ellipticity at 208 nm and 222 nm as a function of pH.

ANS (8-anilino-1-naphthalene sulphonate) is an extrinsic fluorescence probe that is sensitive to polarity. **Figures 2A, B** illustrate how pH affects peroxidase’s ANS fluorescence. At 380 nm, excitation was used to record ANS emission spectra. Proteins exhibit partial ANS binding at pH 2.5. There was a noticeable rise in fluorescence intensity when the pH was further lowered from 2.5 to 1.0. It has been shown in earlier studies that at pH 2 the enzymes displayed their highest ANS binding (Naeem et al., 2015) and the ANS fluorescence intensity increases up to five times compared to the native state the peak of the fluorescence red shifted from 481 nm (pH 2) to 508.05 nm (pH 7). ANS fluorescence decreases on either side of pH 2 and at the alkaline denatured state (UB state, pH 8-12), ANS binding is almost absent and stable up to pH 12.

### 3.4 Circular dichroism

Additionally, the impact of pH variations on peroxidase’s secondary structure was investigated. **Figure 2C** shows the *A. lakoocha* peroxidase CD spectra in acidic, native, and denatured conditions (6 M GuHCl). Strong negative ellipticity is present in the CD spectrum of peroxidase at pH 7 in the far-UV region. This indicates that the molecule may have rich regions of α-helix and β-sheet and be a member of the α + β class of proteins (Manavalan and Jonson, 1983; Kumar et al., 2014). The mean residue ellipticity at 222 nm is -5300.2 deg.cm^2^ dmol^-1^ in the native condition as shown in **Figure 2C**. At pH 1.0, the ellipticity at 222 nm shifts to -3779.9 deg.cm^2^ dmol^-1^. Nevertheless, in the presence of 6 M GuHCl, all the spectrum characteristics of peroxidase disappear under comparable circumstances, suggesting that the enzyme loses its secondary structural organization when denaturing conditions are met. **Figure 2D** illustrates the changes in ellipticity at 208 and 222 nm alter the structure of peroxidase in response to pH. These structural alterations show that, without altering the form of the spectra, the secondary structure contents of proteins stay constant between pH values of 4.0 and 9.5 and decrease on either side of this range. The changes are similar in magnitude at lower pH and in the basic range.

### 3.5 Guanidine hydrochloride induced-unfolding of peroxidase

**Figure 3:**
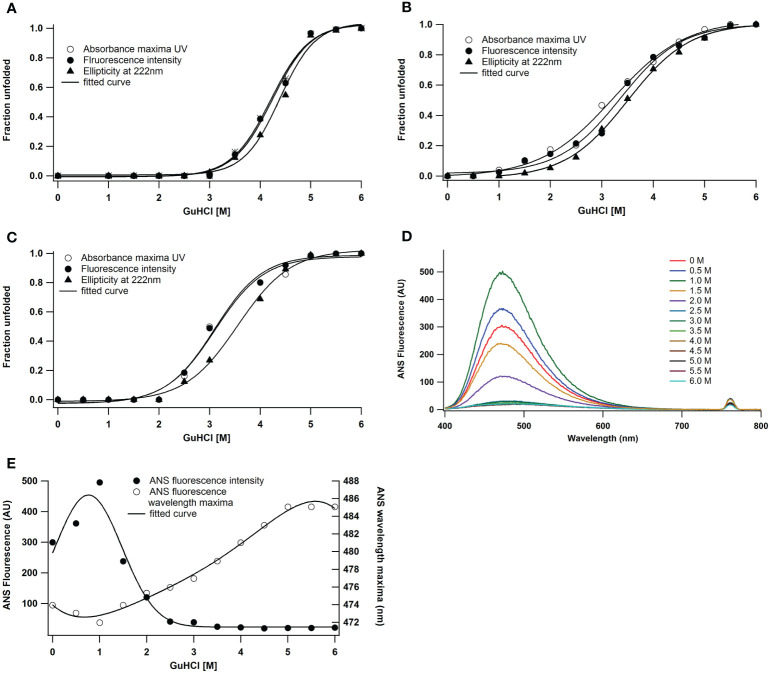
GuHCl induced unfolding of *A lakoocha* peroxidase at different pH: **(A)** pH 7.0, **(B)** pH 2.0, and **(C)** pH 11.0, by following far-UV CD, fluorescence, and UV absorbance, Effect of pH on the ANS fluorescence of *A lakoocha* peroxidase: **(D)** ANS binding spectra, as a function of increasing concentration of GuHCl at pH 2.0 **(E)** ANS fluorescence intensity and wavelength maxima at pH 2.0, as a function of increasing concentration of GuHCl.

Under native conditions, guanidine hydrochloride (GuHCl) caused peroxidase to unfold. Changes in absorbance, fluorescence, and secondary structure revealed structural stability up to 3.0 M GuHCl (**Figure 3A**). In the native state, cooperative sigmoidal curves (**Figure 3A**) with a transition midpoint at 4.5 ± 0.1 M GuHCl are the outcome of GuHCl-induced unfolding. Enzymes, however, showed structural stability at pH 2 up to 1.5 M GuHCl (**Figure 3B**). According to various spectroscopic methods, the transitions are cooperative and non-coincidental (**Figure 3B**). This observation demonstrated that reductions in secondary structure occur after decreases in fluorescence intensity. At pH 2, alterations in intrinsic fluorescence occurred between 1.5 and 5.0 M of GuHCl, while loss in secondary structure happened between 2.0 and 5.0 M. The denaturation transition mid-points (Cm) are 3.5 ± 0.1 M for far UV CD and 3.25 ± 0.1 M for absorbance and fluorescence intensity. Similar research has been done on the impact of GuHCl on a protein’s alkaline denatured condition at pH 11. According to various spectroscopic methods, the transitions are cooperative and coincidental (**Figure 3C**). The enzyme exhibited structural stability until 2.0 M of GuHCl, after which it loses its intrinsic fluorescence and secondary structure between 2.5 and 5.0 M of GuHCl. ANS fluorescence was used to track the denaturation process in both its native and alkaline denatured states, however even after an extended exposure, peroxidase failed to detect any ANS binding. That could be because there was no interaction with the hydrophobic surface. These features suggest that under acidic conditions as opposed to alkaline conditions, *A. lakoocha* peroxidase is more prone to denaturation. Therefore, we studied the molecule’s structural integrity at an acidic pH. The ANS binding pattern of peroxidase at pH 2 is displayed in **Figure 3D** as GuHCl concentration increases. When the molarity of GuHCl was increased, the ANS fluorescence intensity decreased in the acid-denatured condition and its wavelength maximum shifted red (**Figure 3E**).

### 3.6 Urea-induced unfolding of peroxidase

**Figure 4:**
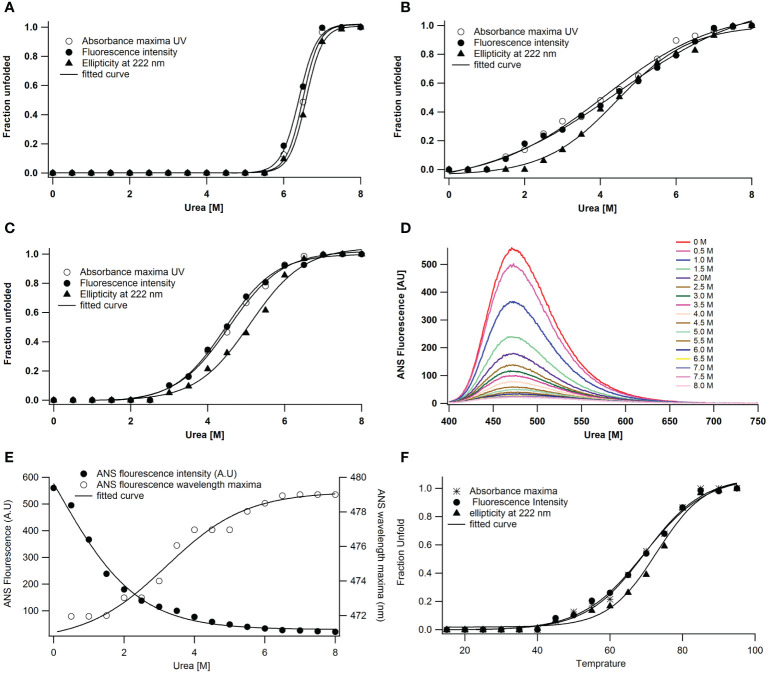
Urea-induced unfolding of *A lakoocha* peroxidase at different pH: **(A)** pH 7.0, **(B)** pH 2.0, and **(C)** pH 11.0, by following far-UV CD, Fluorescence and UV absorbance, Effect of pH on the ANS fluorescence of *A lakoocha* peroxidase: **(D)** ANS binding spectra, as a function of increasing concentration of urea at pH 2.0, **(E)** ANS fluorescence intensity and wavelength maxima at pH 2.0, as a function of increasing concentration of urea, and **(F)** Temperature-induced unfolding of *A lakoocha* peroxidase at pH 7.0: The structural changes are followed by UV absorbance, far- UV CD, and fluorescence.

Since the enzyme maintains its maximum structural and functional characteristics even at 5.5 M urea concentration, urea does not cause any structural disturbance in the protein under neutral conditions (**Figure 4A**). Because enzymes are more vulnerable to denaturants at low and high pH values, these experiments on urea-induced unfolding are conducted at these pH levels. On the other hand, unfolding triggered by urea at a lower pH produced some intriguing results. Fluorescence, absorbance, and far UV CD follow denaturation. The unfolding caused by urea at pH 2 is cooperative but not coincidental (**Figure 4B**). The transition mid-points for the secondary structure are 4.5 ± 0.3 M, while for intrinsic fluorescence and absorbance, they are 4.25 ± 0.1 M. The secondary structure degradation occurred in the range of 2.0 to 7.0 M urea. However, the unfolding transitions by several probes are cooperative at pH 11, where the enzyme is in an alkaline denatured state, with a transition mid-point at 4.75 ± 0.1M (**Figure 4C**).

In the urea-induced unfolding of peroxidase at pH 2, a discrete intermediate state is present, with cooperative but non-coincidental transitions and increased ANS binding at low urea concentrations. The wavelength maximum of peroxidase shifts to red and ANS fluorescence intensity increases at pH 2 (**Figures 4D, E**).

### 3.7 Thermal denaturation of peroxidase

As the most ancient method of denaturing proteins, temperature can alter protein structures and hence reveal a great deal about a protein molecule. **Figure 4F** shows the temperature-induced unfolding of peroxidase at pH 7. The peroxidase temperature-induced unfolding transition midpoints are quite high, ranging from 62.5 ± 0.5°C. Moreover, at 70°C, the protein’s structure dissolves, revealing significant structural agitations already brought on by the higher protein concentration.

## 4 Discussion

Understanding protein structure-function relationships using biophysical techniques is crucial in proteomics (Neet and Lee, 2002). Despite the relevance of Peroxidases, relatively few biophysical investigations on this α + β class of proteins have been conducted. Since catalytic activity depends on pH, an enzyme’s stability dictates its functioning and may prevent it from being used widely (Anfinsen, 1973). To investigate the numerous facets of conformational changes in its structure and the discovery of populated intermediates at extremes of pH, during denaturation, peroxidase’s pH denaturation was therefore tracked using a variety of spectroscopic techniques. The measurement of a sample’s radiation absorption as a function of wavelength or frequency is done using the absorbance technique. These spectra are like the typical heme peroxidase spectra when the protein is in a native state (Sonkar et al., 2015). Peroxidases’ intensely colored prosthetic groups cause absorption bands in the visual spectrum region (α- and β-peaks) and the UV-visible boundary (Soret γ). Additional UV-visible absorption bands include the φ-band at 280 nm, indicating large contribution from tryptophan and tyrosine residues and light absorption by aromatic amino acids in proteins. The peroxidase spectra are stable between pH 2.5 and 8.5, but at lower pH, the heme peroxidase spectra show a left shift with declined absorbance at 404 nm due to denaturation. This indicates a loosely associated form of heme in the acidic range. This study focuses on the shift in absorbance intensity at the 404 nm wavelength, which is a structural change observed in peroxidase. At higher pH, the heme group of peroxidase shows OH^-^ or H_2_O binding, leading to a right shift of the Soret band. The Q-band splits, and the Soret peak shifts to the right. The study reveals that the dissociated form of the heme group and protein denaturation are indicated by a decrease in the peroxidase Soret band at pH 2 and 6 M GuHCl. The proteolytic activity of the enzyme is shown to be preserved in the pH range of 3.5-8.5. This suggests that the native structure and functional stability of the protein are preserved within this pH range. The decrease in proteolytic activity outside a certain range is likely due to the repulsion of columbic forces by the positive charges of polypeptide chains. Fluorescence spectroscopy is a useful tool for studying the behavior of proteins, revealing a red shift in emission maxima when solvent exposure occurs. The quantum yield of fluorescence decreases when chromophores interact with quenching agents, either in the solvent or the protein itself (Dehdasht-Heidari et al., 2021; Raeessi-Babaheydari et al., 2021).The tryptophan excitation spectra show a red shift in emission maxima when chromophores are solvent-exposed, reflecting the tryptophan rich environment. Fluorescence intensity increases at pH 2 but decreases at pH 11, likely due to *A. lakoocha* peroxidase being a heme-containing peroxidase with iron in the heme pocket. The heme acts as a main quencher, quenching energy in the native state. During acid denaturation, tryptophan fluorescence intensity increases due to the distance from the heme group. Further denaturation with 6 M GuHCl enhances the fluorescence intensity as compared to the acidic condition, Thus, as the protein denatures, the donor (tryptophan) and acceptor (here heme) groups move farther apart and hence the intensity increases. Fluorescence wavelength maxima remain unaffected throughout different pH and show very little change in wavelength maxima. Hence, fluorescence intensity was used to reveal the changes at different pH. The pH-induced transition of peroxidase in the alkaline range is cooperative. The transition from the native state to the acid unfolded state occurs between pH 4.0 and 2.0. Fluorescence intensity decreases with higher pH, possibly due to the reduced distance between tryptophan and specific quenching groups like protonated carboxyl, protonated imidazole, deprotonated amino groups, and tyrosinate. This results in the quenching of tryptophan fluorescence (Dehdasht-Heidari et al., 2021; Raeessi-Babaheydari et al., 2021).

The measurement of hydrophobic surface exposure in enzymes is done using an approach called ANS binding, which determines the structural integrity of a protein. When *A. lakoocha* peroxidase was exposed to pH values higher than 3, no ANS binding was seen. Nonetheless, some binding was observed at lower pH values, but to a lesser extent. A blue shift of the emission maxima and an increase in fluorescence intensity were indicative of an increase in ANS binding as the pH dropped from 3 to 2.5. When pH is lowered further, the protein is refolded at pH 0.5, which forces the molecule into an acid-refolded form due to the counterions’ protection of columbic repulsions. However, at pH 2, this protein exhibits maximal ANS binding, a sizable amount of secondary structure, and no tertiary connections. This abrupt increase in ANS fluorescence intensity is caused by hydrophobic surfaces being exposed to their maximum, suggesting that a molten globule condition is present, at pH 2. These experimental findings demonstrate that the peroxidase maintains both its native structure and proteolytic activity within the pH range of 3.0-10.0. This is supported by the lack of ANS binding within this range, which suggests that the protein has a relatively rigid and stable native conformation over a wide pH range but becomes more flexible and sensitive to changes in acidity. The first transition phase, which occurs between pH 2.5 and 4.0 (midpoint 3.25), and the second transition, which occurs between pH 1 and 2.5 (midpoint 1.5), are both seen in the pH-induced conversion of this protein. According to absorbance and fluorescence spectra, proteins unfold to create acid-unfolded states (UA) at pH 1.5 or lower, while at pH 7.5 and higher, they become an alkaline denatured state (UB). The UA state is then further lowered to pH 0.5, which causes refolding; this new state is known as the acid-refolded “A-state”. The structure and stability of *A. lakoocha* peroxidase were further investigated using hydrophobic dye ANS and circular dichroism (CD) spectroscopy. The enzyme’s Far-UV CD spectra revealed that it may have separate α and β-rich domains and is consistent with the α + β family of proteins. The peroxidase was found to be quite stable in terms of secondary structure, with only a 50% decrease in ellipticity at lower pH. However, under denaturing conditions, the peroxidase lost all prominent peaks in aromatic regions, indicating the loss of secondary structural features and the enzyme being in an unfolded state. It was discovered that the changes in ellipticity at different pH values followed three intermediate forms. The first shift from the native state to the acid unfolded state happened between pH 4 and 1, and the half bell shape in the pH 1-4 range showed the disappearance of certain significant peaks. The diminished activity and loss of secondary structure are indicative of the unfolded state of the enzyme acid. Under natural conditions, the second transition happened between pH 4 and 8, while the third transition happened between pH 8 and 12, with a midpoint at pH 10.5. Using the hydrophobic dye ANS further showed that a significant number of hydrophobic clusters were still present in the acid-unfolded form even after the secondary structure had melted. Information regarding the conformational stability of the enzyme can be obtained by analyzing denaturation curves brought on by GuHCl-induced unfolding of peroxidase. GuHCl desaturates peroxidase in two states at native pH; values were determined using the two-state model, with ΔG of 4.2 kcal moL^−1^ M^−1^and a high transition mid-point of around 4.5 ± 0.1M. On the other hand, at pH 2, the enzyme unfolds cooperatively. Here the thermodynamic parameters, which are derived from three-state models and presented in Equations from the “Materials and Methods” section, are applied. The transition midpoints of the secondary and tertiary structures occur at 3.5 ± 0.1 M and 3.25 ± 0.1 M GuHCl, respectively, and the ΔG values are 2.9 and 3.1 kcal moL^−1^ M^−1^, with cooperativity index values of 0.82 and 0.95 kcal moL^−1^ M^−1^(**Table 1**). At pH 11.0, the transition mid-points are lower with ΔG of 1.1 kcal moL^−1^ M^−1^ (Dwevedi et al., 2010). ANS fluorescence, a crucial tool in tracking denaturation processes, exhibits binding in acidic conditions, where a red shift in the wavelength maximum and corresponding decrease in intensity occur. The maximum ANS binding and other spectroscopic features in heme-containing peroxidase occur at pH 2 due to an increase in distance from the heme group, which acts as a quencher. This action is also documented in lactose peroxidase and horse radish peroxidase (Carvalho et al., 2003; Zelent et al., 2010). Since no ANS binding was seen, the absence of surface hydrophobic patches is suggested by ANS binding to peroxidase at pH 2 in the presence of ≥2.5M GuHCl. One intermediate was discovered at 1.0 M GuHCl while the concentration of GuHCl was being reduced. At this point, the protein exhibits the maximum ANS fluorescence intensity (**Figure 3E**), indicating the exposure of hydrophobic residues in this state. That makes sense given the molten globule state (MG) theory at that specific GuHCl concentration. The lack of ANS binding to peroxidase at concentrations of 3 M GuHCl and higher indicates the absence of hydrophobic patches on the surface as well as refolding and stabilization brought on by stabilizing interactions between the intermediate state and GuHCl.

**Table 1:** Unfolding parameters of *A. lakoocha* peroxidase.

Condition	Denaturant	Method	Transition mid-point	ΔG (H2O) kcal/mol	*m* *(kcal mol^-1^ M^-1^)*
**pH 7.0** **(Native state)**	GuHCl	CD[Θ]_222_ Fluorescence absorbance	4.5 ± 0.1 M 4.5 ± 0.1 M 4.5 ± 0.1 M	4.2 ± 0.1 4.1 ± 0.1 4.5 ± 0.2	0.93 ± 0.1 0.91 ± 0.2 1 ± 0.1
	Urea	CD[Θ]_222_ Fluorescence absorbance	6.5 ± 0.1 M 6.5 ± 0.1 M 6.5 ± 0.1 M	5.2 ± 0.1 4.5 ± 0.1 4.7 ± 0.1	0.8 ± 0.1 0.69 ± 0.1 0.72 ± 0.1
**pH 2.0** **(Molten globule state)**	GuHCl	CD[Θ]_222_ Fluorescence absorbance	3.5 ± 0.1 M 3.25 ± 0.1 M 3.25 ± 0.1 M	2.9 ± 0.1 3.1 ± 0.1 2.8 ± 0.1	0.82 ± 0.1 0.95 ± 0.1 0.86 ± 0.1
	Urea	CD[Θ]_222_ Fluorescence absorbance	4.5 ± 0.3 M 4.25 ± 0.1 M 4.25 ± 0.1 M	2.5 ± 0.3 2.7 ± 0.1 2.1 ± 0.1	0.55 ± 0.1 0.63 ± 0.1 0.49 ± 0.1
**pH 11.0** **(Alkaline denatured state)**	GuHCl	CD[Θ]_222_ Fluorescence absorbance	3.5 ± 0.1 M 3.5 ± 0.1 M 3.5 ± 0.1 M	1.5 ± 0.1 1.1 ± 0.1 1.2 ± 0.1	0.42 ± 0.1 0.31 ± 0.1 0.34 ± 0.1
	Urea	CD[Θ]_222_ Fluorescence absorbance	4.75 ± 0.1 M 4.75 ± 0.1 M 4.75 ± 0.1 M	1.7 ± 0.1 1.6 ± 0.1 1.8 ± 0.1	0.35 ± 0.1 0.33 ± 0.1 0.37 ± 0.1

The stability of the enzyme peroxidase against urea was evaluated under different pH conditions. The enzyme was stable against urea, as evidenced by the fact that it maintained its original structure and function at native pH even after being exposed to 5.5M urea with ΔG of 5.2 kcal mol-1 M-1. These values were obtained using a two-state model. The thermodynamic parameters, on the other hand, are obtained using three-state models at an acidic environment (pH 2). The enzyme unfolded cooperatively at pH 2, where urea caused the secondary and tertiary structures’ transition midpoints to occur at 4.5 ± 0.3M and 4.25 ± 0.1M urea, respectively. The corresponding ΔG values were 2.5 and 2.7 kcal moL^−1^ M^−1^, with cooperativity index values of *m_1_* and *m_2_* being 0.55 and 0.63, respectively (Dubey and Jagannadham, 2003). Moreover, the unfolding process was investigated using Far-UV CD and fluorescence; the outcomes demonstrated cooperative and non-overlapping transitions as well as an increase in ANS binding at low urea concentrations. In addition, an increase in urea concentration was accompanied by a decrease in the intensity of ANS fluorescence, which peaks at pH 2 in the absence of denaturant. The sigmoidal variation in fluorescence intensity with urea at pH 2 is caused by configurational changes in the amino acids. The unfolding process at pH 2 revealed wavelength maxima and far-UV CD alterations like those at pH 7. Disruption of the enzyme’s functional structure was responsible for a total loss of activity above 5 M urea, a sigmoidal rise in fluorescence intensity, ANS binding, and conversion to beta-sheet-like spectra observed by far-UV CD above 3.5 M urea. These findings imply the existence of a distinct intermediate stage in the peroxidase unfolding caused by urea.

In summary, this investigation revealed that in comparable circumstances, the mode in which enzyme peroxidase reacts to urea and guanidine hydrochloride (GuHCl) varies. According to the findings, urea is less efficient than GuHCl at lowering the mid-points of unfolding at a particular concentration. The ionic nature of the two denaturants is most likely the cause of this behavioral variation. Despite having distinct hydrophobic and electrostatic characteristics, urea and GuHCl interact with peptide bonds. Unlike urea, which lacks this function, positively charged GuHCl may stabilize the protein by its anionic impact. Furthermore, GuHCl’s counter-anion, chloride, may block electrostatic repulsion and permit forces that promote protein folding and stability. It is possible that GuHCl’s stabilizing impact is not primarily ionic (Zarrine-Afsar et al., 2006).

The effects of temperature on *A. lakoocha* peroxidase structure and activity were also covered in the study. The enzyme was found to be highly stable and retained around 45% of its structure under native circumstances. The transition midpoint (Tm) was determined to be 62.5 ± 0.5°C, with ΔG of 4.9 kcal moL^−1^ M^−1^, ΔSm 178.5 *Cal/mol/deg* and ΔHm 64.5 kcal moL^−1^ lying within the range as previously reported (Dwevedi et al., 2010; Patel and Jagannadham, 2013). ΔG values are comparable with the 4.2 kcal/mol ΔG _(H2O)_ obtained by GuHCl denaturation of *A. lakoocha* peroxidase at pH 7.0. It was discovered that the enzyme was highly stable and retained around 45% of its structure under neutral circumstances. This presentation of the accumulation of intermediates in the urea and GuHCl-induced unfolding routes suggests that peroxidase may have two domains that exhibit distinct behaviors under different denaturing conditions, resulting in the production of intermediate states.

## 5 Conclusion

Utilizing various spectroscopic methods and varying temperatures, denaturing conditions, and pH, we discovered three distinct folding states of peroxidase from the latex of *A. lakoocha*. With an entire secondary structure, hydrophobic patches absent, and proteolytic activity, the enzyme is in its natural state at pH 7. The enzyme goes through intermediate phases like the molten globule state, the acid-unfolded state (UA state), and the acid-refolded state (A state) at lower pH values. *A. lakoocha* peroxidase is more prone to denaturation in acidic environments than in alkaline ones, as evidenced by the nearly nonexistent ANS binding in the alkaline denatured state (UB state). Tryptophan fluorescence is quenched at higher pH values in the UB state because of a shorter distance between tryptophan and certain quenching groups. When exposed to GuHCl concentrations greater than 5 M, the enzyme can undergo denaturing conditions and eventually become denatured. GuHCl exhibits anionic behavior at low concentrations (1.0 M) and chaotropic behavior at concentrations of 2.5 M and above. Subsequent tests showed that the peroxidase lost all its secondary structure when it was incubated in 8 M urea for an entire night, but only 60% of it when it was incubated in 6 M GuHCl. Our research indicates that the refined peroxidase found in *A. lakoocha* latex is a reliable model system for investigating protein folding pathways. The discovery of these intermediate states has advanced the field of protein folding significantly and provides a basis for further investigation into the physiological substrates, structural features, and possible uses of the enzyme in the biotechnology and pharmaceutical industries.

## Data availability statement

The original contributions presented in the study are included in the article/supplementary material. Further inquiries can be directed to the corresponding author.

## Author contributions

KS: Conceptualization, Data curation, Formal analysis, Investigation, Methodology, Validation, Writing – original draft. MP: Writing – review & editing. AK: Formal analysis, Software, Writing – review & editing. HC: Writing – original draft, Writing – review & editing. MJ: Supervision, Writing – review & editing.
